# Beyond numbers: The governance role of data assets in real earnings management

**DOI:** 10.1371/journal.pone.0351687

**Published:** 2026-07-07

**Authors:** Miao Zhang, Huaian Qian, Tengfei Ma

**Affiliations:** 1 School of Finance, Jiangsu Vocational College of Finance and Economics, Jiangsu, China; 2 School of Accounting, Jiangsu Vocational College of Finance and Economics, Jiangsu, China; 3 School of Accounting, Dongbei University of Finance and Economics, Liaoning, China; Shanghai Business School, CHINA

## Abstract

While data assets are increasingly recognized as strategic resources in the digital economy, their implications for financial reporting quality remain underexplored. Employing a textual analysis approach based on deep learning techniques to construct a measure of data assets, this study investigates their impact on real earnings management (REM) using a sample of Chinese A-share listed firms from 2010 to 2023. We document a negative association between data assets and REM. Our findings remain robust after addressing endogeneity concerns using instrumental variables based on regional digital infrastructure and propensity score matching. Mechanism analyses reveal that data assets mitigate REM through two distinct channels: curbing managerial short-termism and strengthening external monitoring via increased analyst coverage. Cross-sectional tests indicate that this governance effect is more pronounced among firms facing earnings pressure. Furthermore, data assets primarily constrain cost-side manipulation strategies, specifically abnormal production costs and discretionary expenditures. Finally, we show that the reduction in REM driven by data assets contributes to enhanced long-term firm value. Collectively, these findings uncover a novel governance effect of data assets, suggesting that digital infrastructure investments yield positive externalities for corporate financial behavior.

## 1 Introduction

In the digital economy, data have evolved from an operational by-product into a strategic resource that reshapes value creation and corporate competition. Data assets differ from traditional tangible assets such as land or machinery. They are largely non-rivalrous, inexpensive to replicate, and often exhibit increasing returns to scale [1]. These attributes allow firms to improve decision quality, forecast demand and risk more accurately [2]. Recognizing this shift, regulators have begun to formalize the role of data in financial reporting. For instance, China’s Ministry of Finance issued the *Interim Provisions on Accounting Treatment of Enterprise Data Resources* in 2023. Following the introduction of this policy, some firms have voluntarily disclosed information of data assets in annual reports and regulatory filings to mitigate information asymmetry and facilitate price discovery [3,4].

While extensive literature examines how digital transformation affects firm performance or valuation [5,6], the implications of data assets for financial reporting quality remain underexplored. This study focuses on real earnings management (REM), a practice where managers deviate from optimal business strategies to meet earnings thresholds [7]. Compared with accrual-based earnings management, REM alters actual business activities and is therefore more opaque and more difficult for auditors and investors to detect. Although REM can generate short-term reporting benefits, extensive evidence suggests that it harms long-term performance [8]. Given the concealment advantages of REM and the limitations of traditional monitoring, identifying effective constraints on such behavior is both theoretically and practically important.

By distinguishing data assets from IT investments, we argue that data assets are an important complement to traditional governance mechanisms by reshaping the corporate information environment. Unlike general investments in IT infrastructure to facilitate interconnectivity, data assets reflect the accumulation, integration, and use of granular operational information in the context of the business process [9]. We propose two channels through which data assets discipline REM. As regards internal monitoring, data assets create a verifiable digital record of operational activities [10]. This will significantly reduce the monitoring costs that typically limit the suppression of opportunistic behavior and thus reduce the asymmetry of information on which REM is based. Meanwhile, the substantial investment in data assets provides a credible signal of long-term value creation [11]. This signals to the market that management prioritizes sustainable growth, effectively relieving short-term earnings pressure. Moreover, this focus on sustainability attracts long-term investors who discipline managerial behavior through their monitoring activities [12,13].

Using a sample of A-share listed companies on the Shanghai and Shenzhen Stock Exchanges from 2010 to 2023, we empirically examine the relationship between data assets and REM. We use a textual analysis methodology to construct a measure of data assets based on the frequency of specific keywords in annual reports. This approach allows us to capture the accumulation of data resources rather than merely IT expenditure. Our findings indicate a significant negative relationship between data assets and REM. To address potential endogeneity concerns, we apply instrumental variable (IV) methods and propensity score matching (PSM), with results remaining robust across both methodologies. Furthermore, we explore the mechanisms through which data assets exert their influence. Our analysis suggests that data assets mitigate REM primarily by reducing managerial short-termism and by enhancing analyst coverage, which strengthens external monitoring and promotes information transparency. Further analysis reveals that the mitigating effect of data assets on REM is more pronounced among firms facing greater earnings pressure. We also decompose REM into its components to identify specific channels of influence. The results indicate that data assets significantly reduce earnings manipulation related to production costs and discretionary expenditures, both of which are commonly used cost-side strategies. Finally, we demonstrate that the reduction in REM driven by data asset utilization is associated with improved firm value, underscoring the broader role of data governance in fostering sustainable corporate performance and long-term value creation.

This study makes several contributions to the literature.

First, we extend the growing body of literature on the economic consequences of data assets by exploring their impact on REM. While prior studies have primarily emphasized how data assets enhance firm performance, drive innovation, and improve market efficiency, we provide evidence that data assets perform a pivotal monitoring function that curbs opportunistic managerial behavior. By distinguishing data assets from IT investments, our study shows their unique capacity to deter REM, thus revealing a new channel through which data-driven infrastructures advance the quality of financial reporting and support the long-term sustainability of companies.

Second, we extend the literature regarding the determinants of REM. While prior studies have concentrated on traditional governance factors such as board composition and ownership structure, we shift the focus toward the influence of data assets. We complement this literature by illuminating how data assets serve as a strategic instrument that modulates managerial reporting decisions. Our findings suggest that in the digital economy, effective governance relies not only on conventional organizational structures but also on the granularity of the corporate information environment.

Third, our study empirically endorses the regulatory push for recognition of data asset. We show that capitalizing and disclosing data assets yields positive externalities that enhance governance and market efficiency. Our findings reveal that these policies are not simply administrative hurdles but are vital for mitigating information asymmetry. By establishing that data assets effectively deter earnings management, we argue for the accelerated adoption of comprehensive disclosure frameworks.

## 2 Literature review and hypothesis development

### 2.1 Literature review on real earnings management

The scope of earnings management research has evolved from the original focus on discretionary accruals to investigations of real activities manipulation [14]. While Accrual-based Earnings Management (AEM) relies on adjusting accounting estimates within the bounds of GAAP, REM involves directly altering actual operational activities, making it much more difficult to detect. Consequently, REM is often preferred in strict regulatory environments due to its opacity [15]. As regulatory oversight and financial reporting standards tighten, managers increasingly shift from AEM to REM, leveraging it as a more flexible and covert mechanism for earnings manipulation [16–18].

The motivations behind REM are usually interpreted from two competing theoretical perspectives: the agency theory and the signaling theory [8,19]. The prevailing view in existing literature is that REM stems primarily from managerial opportunism driven by agency conflicts. Given that compensation and job security are often tied to short-term stock performance or earnings targets, managers tend to sacrifice long-term shareholder value to achieve immediate results [20]. Under pressure to meet or beat analyst forecasts, avoid reporting losses, or sustain earnings growth, managers are prone to managerial myopia [21]. Conversely, the signaling perspective argues that managers utilize REM to convey private information regarding a firm’s future potential to the market [8].

Recent governance-oriented studies have highlighted the role of structural factors in mitigating agency costs. Li et al. (2025) indicates that optimizing board and ownership structures, combined with strong eco-technology, can enhance sustainability and circular economy results. This implies that a sound governance system is essential to aligning management decisions with long-term interests [22]. Similarly, Wu et al. (2025) underscores the synergy between ownership structure and financing strategies in driving innovation and environmental fulfillment [23]. However, the efficacy of these structural factors is heavily contingent upon the transparency of corporate information. REM remains a persistent challenge because operational decisions often fall within the realm of “business judgment,” rendering them opaque to traditional oversight mechanisms [24].

### 2.2 Literature review on data assets

Data assets are data resources owned or controlled by an enterprise that are recorded in electronic or other forms and promise future economic benefits [1,2]. This concept goes beyond the mere accumulation of raw data; it implies the systematic integration and use of data for the purpose of creating value. The early literature on the business value of information technology focused to a large extent on tangible IT investments [5]. However, recent scholarship distinguishes data assets as a specific form of digital capital that generates value through information services rather than infrastructure ownership [9,25]. Unlike traditional physical assets, data assets have a non-rivalry character, which means they can be used simultaneously across multiple algorithms and business lines without any fragmentation [26]. In the meantime, data accumulation often exhibits increasing returns to scale, allowing firms to reduce future uncertainty and maintain long-run growth [27,28].

The utilization of data assets has been linked to various positive economic outcomes. Hu et al. (2022) finds that data assets significantly enhance operational efficiency by optimizing resource allocation [9]. In terms of corporate growth, Zheng et al. (2025) shows that data assets spur corporate R&D investment by improving information integration [10], while Li et al. (2024) finds that data assets further drive sustainable development, evidenced by improved ESG performance [11]. In the context of capital markets, Sun and Du (2024) argue that data asset disclosure reduces stock price synchronicity, facilitating better price discovery [3]. Furthermore, Qian et al. (2025) finds that such disclosure reduces information asymmetry in debt markets, directly aiding firms in acquiring bank lending [29].

### 2.3 Hypothesis development

The relationship between data assets and REM can be theoretically based on the integration of agency theory, information asymmetry theory, and the resource-based view. REM tends to thrive in environments with high information opacity and limited monitoring [24,30]. This is because external stakeholders and even internal boards often lack granular, real-time information needed to distinguish between necessary strategic adjustments and value-destroying manipulations [14].

Data assets change the information environment within a firm. Compared to traditional physical assets, data assets generate, process and disseminate information in real time [1]. The agency theory states that monitoring provides a method to control behavior that involves opportunism [17]. Data assets reduce monitoring costs by creating a digital record of activities performed. The integration of financial systems with real-time monitoring tools allows for continuous auditing and oversight [31,32]. For example, the use of IOT technologies that interconnect devices greatly enhances supply chain transparency. This reduction in opacity limits areas where manipulative activities may remain hidden [33]. When operational metrics relating to operations that indicate inventory levels and production costs appear in real-time [7], the detection risk for managers attempting to manipulate these activities increases substantially [34].

Moreover, data assets strengthen internal governance mechanisms. Cheng et al. (2016) highlights that key subordinate executives play a crucial role in monitoring the CEO [35]. However, the effectiveness of this monitoring depends on access to high-quality information. Data assets democratize information access within the executive team. Research indicates that big data quality and diagnosticity significantly improve firm decision quality [36]. When data functions as a shared organizational asset, executives gain objective, data-driven evidence. This empowers them to challenge suboptimal CEO directives, realign managerial actions with the firm’s long-term interests, and reduce the myopic focus that often drives R&D cuts [12].

Beyond the monitoring perspective, the resource-based perspective suggests that data assets improve firm capabilities, which reduces the need for REM. A primary driver of earnings manipulation is the pressure to close the gap between actual performance and market expectations [18]. Data assets help to close this gap by driving genuine improvements. Ma et al. (2024) provides evidence that data assets are useful in optimizing the cost behavior of firms, thus improving operational efficiency [37]. Furthermore, big data analytics enhance dynamic capabilities, allowing firms to adapt to market changes more effectively [36]. When a firm can achieve earnings targets through efficiency gains and successful innovation facilitated by data assets [9,10], the pressure to resort to value-destroying manipulation diminishes.

Finally, data assets enhance external monitoring through the signaling channel. Disclosure and possession of data assets signal to the capital market that the firm maintains superior information processing capabilities and a long-term strategic orientation. Sun and Du (2024) find that disclosure of data assets significantly reduces stock prices synchronicity and facilitates better price discovery [3]. Li et al. (2024) finds that data assets drive corporate sustainable development, signaling a commitment to long-term value creation [11]. As the external information environment becomes more transparent, external stakeholders can scrutinize the firm operations more effectively, as higher coverage is linked to lower earnings management [38]. Based on these arguments, we propose the following hypothesis:

H1: Data assets are negatively associated with the extent of real earnings management.

## 3 Research design

### 3.1 Sample selection

We select A-share listed companies on the Shanghai and Shenzhen Stock Exchanges from 2010 to 2023 as our initial sample. To refine the dataset, several filtering procedures are applied. First, firms operating in the financial and insurance sectors are excluded due to their distinct financial reporting structures, which differ significantly from those of other industries. Second, firms classified as *ST or ST statuses are eliminated, as their operational conditions and strategic decisions often diverge from those of normally operating firms. Third, observations with missing values are removed to maintain data integrity. Additionally, to mitigate the influence of outliers, all continuous variables are winsorized at the 1st and 99th percentiles. This procedure ensures that statistical inferences are not driven by extreme values inherent in financial ratios. All firm-level data are obtained from the China Stock Market & Accounting Research (CSMAR) Database.

### 3.2 Key variable definitions

#### 3.2.1 Real earnings management.

Following Roychowdhury (2006) [7], we first estimate the abnormal levels of production costs (*Abprod*), cash flow from operations (*Abcfo*), and discretionary expenses (*Abdisx*). Each abnormal indicator is derived from the residuals of its corresponding estimation model, as specified below:


$$\begin{array}{c} \frac{PROD_{it}}{TA_{it-\text{1}}}={\alpha_{\text{0}}+\alpha }_{\text{1}}\frac{\text{1}}{TA_{it-\text{1}}}+\alpha_{\text{2}}\frac{SALES_{it}}{TA_{it-\text{1}}}+\alpha_{\text{3}}\frac{\Delta SALES_{it}}{TA_{it-\text{1}}}+\alpha_{\text{4}}\frac{\Delta SALES_{it-\text{1}}}{TA_{it-\text{1}}}+\varepsilon_{it} \end{array}$$ (1)

$$\begin{array}{c} \frac{CFO_{it}}{TA_{it-\text{1}}}=\alpha_{\text{0}}+\alpha_{\text{1}}\frac{\text{1}}{TA_{it-\text{1}}}+\alpha_{\text{2}}\frac{SALES_{it}}{TA_{it-\text{1}}}+\alpha_{\text{3}}\frac{\Delta SALES_{it}}{TA_{it-\text{1}}}+\varepsilon_{it} \end{array}$$ (2)

$$\begin{array}{c} \frac{{DISX}_{it}}{TA_{it-\text{1}}}={\alpha_{\text{0}}+\alpha }_{\text{1}}\frac{\text{1}}{TA_{it-\text{1}}}+\alpha_{\text{2}}\frac{SALES_{it-\text{1}}}{TA_{it-\text{1}}}+\varepsilon_{it} \end{array}$$ (3)

Where *PROD*_*it*_ denotes the production costs of firm *i* in year *t*; *CFO*_*𝑖𝑡*_ represents the operating cash flow of firm *i* in year *t*; *DISX*_*it*_ denotes the discretionary expenses; *TA*_*it-1*_ is total assets at the end of year *t-1*; *SALES*_*it*_ measures net sales in year *t*; and *ΔSALES*_*it*_ is the change in net sales from year *t-1* to year *t*.

REM typically manifests through abnormally high production costs, abnormally low operating cash flows, and abnormally low discretionary expenses. Recognizing that firms may employ multiple REM strategies simultaneously, we construct a composite measure of REM (*REM*) by aggregating these three abnormal components, as defined in Eq (4).


$$\begin{array}{c} REM=Abprod-Abcfo-Abdisx \end{array}$$ (4)

#### 3.2.2 Data assets.

Although the Ministry of Finance’s 2023 *Interim Provisions* authorize the recognition of data resources as assets starting in 2024, relying on financial data for measurement remains impractical for empirical research. As of the 2024 reporting period, only approximately 100 A-share listed companies have recognized data resources in their financial statements, leading to severe sample selection bias and a lack of comparability. Therefore, to capture the substantive utilization of data assets across a broad sample, we adopt a textual analysis approach. Drawing on the definition provided in the White Paper (Version 6.0) (The Data Asset Management Practice White Paper (Version 6.0) was jointly issued by the Big Data Technology and Standard Committee and the China Academy of Information and Communications Technology (CAICT) in June 2023.), we select “information,” “network,” “digital,” and “data” as seed words. We then employ the Word2Vec neural network model and deep learning techniques to identify sets of semantically similar words for each seed term. To ensure semantic accuracy within a corporate context, the model is trained on a corpus comprising the annual reports of all A-share listed companies. Finally, words with the highest cosine similarity scores are retained and manually screened to ensure thematic relevance and validity.

Subsequently, we calculate the frequency of these seed words and their semantically related counterparts in firms’ annual reports. Our final measure of data assets, denoted as *DA*, is defined as the natural logarithm of the total frequency count. This variable serves as a proxy for the intensity of data asset accumulation and deployment. The final dictionary of keywords used to construct the data assets measure is provided in S1 Table.

### 3.3 Model specification

We assess whether data assets mitigate REM by estimating the following baseline regression model:


$$\begin{array}{c} {REM}_{it}=\alpha_{\text{0}}+\alpha_{\text{1}}{DA}_{it}+\alpha_{\text{2}}{Controls}_{it}+Year+Industry+\varepsilon_{it} \end{array}$$ (5)

In this specification, *REM* denotes the level of REM for firm *i* in year *t*. The key explanatory variable, *DA*, captures the firm’s investment in and utilization of data assets. We control for a set of firm-specific characteristics that may influence REM outcomes. *Size* is measured as the natural logarithm of total assets. *Lev* represents the capital structure, calculated as total liabilities divided by total assets. *Roa* denotes return on assets, computed as net income divided by total assets. *Growth* is the year-over-year percentage change in operating revenue.

We also incorporate a set of governance-related variables. *Dual* is a dummy variable equal to 1 if the CEO concurrently serves as the board chair, and 0 otherwise. *Soe* is a dummy variable equal to 1 if the firm is state-owned. *Top1* represents the ownership share held by the largest shareholder, capturing ownership concentration. *Big4* is a dummy variable equal to 1 if the firm’s auditor is one of the Big Four accounting firms. *Indep* measures the proportion of independent directors on the board, while *Board* is defined as the natural logarithm of the total number of directors serving on the board. *Mshare* reflects the level of managerial ownership, and *Mpay* captures the total compensation of the top three executives. *AEM* controls for accrual-based earnings management. *Year* and *Industry* fixed effects are included to account for unobserved time-specific and sector-specific factors. Detailed variable definitions are provided in Table 1.

**Table 1:** Variable definitions and calculation.

Variable Type	Variable	Definition	Calculation
Dependent Variable	*REM*	Real earnings management	Composite index combining *Abprod*, *Abcfo*, and *Abdisx*
Independent Variable	*DA*	Data assets	Natural logarithm of the frequency of data-related keywords in annual reports
Control Variables	*Size*	Firm size	Natural logarithm of total assets
	*Lev*	Financial leverage	Total liabilities divided by total assets
	*Roa*	Return on assets	Net income divided by total assets
	*Growth*	Revenue growth rate	Annual percentage change in operating revenue
	*Dual*	CEO duality	Equals 1 if the CEO also serves as the board chair, 0 otherwise
	*Soe*	State ownership	Equals 1 if the firm is state-owned, 0 otherwise
	*Top1*	Ownership concentration	Shareholding percentage of the largest shareholder
	*Big4*	Big Four auditor	Equals 1 if audited by a Big Four accounting firm, 0 otherwise
	*Indep*	Board independence	Proportion of independent directors on the board
	*Board*	Board size	Natural logarithm of the total number of directors on the board
	*Mshare*	Managerial ownership	Percentage of shares held by executives
	*Mpay*	Executive compensation	Total compensation paid to the top three executives
	*AEM*	Accrual-based earnings management	Estimated using the modified Jones model

## 4 Empirical results

### 4.1 Descriptive statistics

Table 2 reports the descriptive statistics. The mean value of *REM* is −0.027, with a standard deviation of 0.224; the interquartile range spans from −0.123 to 0.104, and the maximum reaches 0.524, indicating that a non-negligible number of firms engage in REM.

**Table 2:** Summary statistics.

Variable	N	Mean	SD	p25	p50	p75	Max
*REM*	20770	−0.027	0.224	−0.123	−0.001	0.104	0.524
*DA*	20770	3.276	0.696	2.890	3.178	3.555	6.979
*Size*	20770	22.550	1.275	21.630	22.360	23.300	26.180
*Lev*	20770	0.428	0.194	0.273	0.423	0.573	0.891
*Roa*	20770	0.052	0.056	0.021	0.046	0.080	0.214
*Growth*	20770	0.190	0.359	0.004	0.126	0.286	2.129
*Dual*	20770	0.284	0.451	0	0	1	1
*Soe*	20770	0.349	0.477	0	0	1	1
*Top1*	20770	34.470	14.660	22.990	32.330	44.390	73.130
*Big4*	20770	0.074	0.261	0	0	0	1
*Indep*	20770	37.520	5.325	33.330	35.710	42.860	57.140
*Board*	20770	2.134	0.195	1.946	2.197	2.197	2.639
*Mshare*	20770	13.450	18.910	0.004	1.078	25.340	66.560
*Mpay*	20770	14.710	0.716	14.230	14.680	15.150	16.560
*AEM*	20770	0.009	0.089	−0.031	0.011	0.055	0.257

Note: This table reports the summary statistics for the main variables. The sample consists of 20770 firm-year observations spanning the period from 2010 to 2023.

The independent variable *DA* has a mean of 3.276 and a standard deviation of 0.696. The 75th percentile and the maximum value are 3.555 and 6.979, respectively. The right-skewed distribution of *DA* suggests that while many firms maintain moderate levels of data asset investment, a subset exhibits significantly higher engagement. This pattern is consistent with innovation diffusion theory, which posits that early adopters leverage emerging technologies ahead of their peers, providing a robust empirical context for examining the impact of data assets on REM.

### 4.2 Hypothesis tests

**Fig 1:**
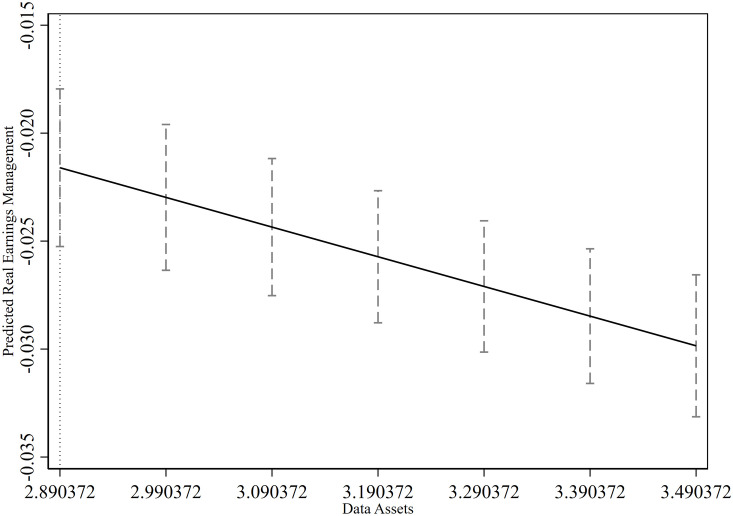
Marginal effect of data assets on real earnings management. Note: This figure illustrates the predicted level of real earnings management across the interquartile range of data assets, with 95% confidence intervals.

Table 3 presents the main regression results. In Column (1), which controls only for year and industry fixed effects, the coefficient on DA is significantly negative (−0.014, t = −4.917). This negative association becomes even stronger and remains robust (−0.017, t = −7.595) after incorporating firm-level control variables in Column (2). Beyond statistical significance, we further assess the economic magnitude of this effect by examining the sensitivity of *REM* to an interquartile shift in *DA*. Given that the interquartile range of *DA* is 0.665, an increase in data assets from the 25th to the 75th percentile corresponds to a reduction in *REM* of approximately 0.011, holding other factors constant. Relative to the sample standard deviation of *REM*, this reduction represents approximately 5.0%, indicating a substantial economic impact. Consistent with these calculations, Fig 1 plots this marginal effect, illustrating a distinct and monotonic downward trend in *REM* as data asset accumulation increases.

**Table 3:** Main regression results.

Variables	(1)	(2)
	REM	REM
*DA*	−0.014***	−0.017***
	(−4.917)	(−7.595)
*Size*		0.030***
		(19.275)
*Lev*		0.015
		(1.601)
*Roa*		−2.295***
		(−66.131)
*Growth*		0.077***
		(12.68)
*Dual*		−0.013***
		(−4.513)
*Soe*		0.002
		(0.533)
*Top1*		−0.000*
		(−1.863)
*Big4*		−0.047***
		(−9.005)
*Indep*		−0.001***
		(−2.923)
*Mshare*		0.000
		(0.456)
*Board*		−0.027***
		(−3.293)
*Mpay*		−0.043***
		(−17.184)
*AEM*		1.054***
		(53.234)
Constant	0.017	0.068
	(0.959)	(1.578)
N	20770	20770
Adj. R2	0.006	0.387
Year FE	YES	YES
Industry FE	YES	YES

Note: This table examines the association between data assets and REM. The sample consists of 20,770 firm-year observations from 2010 to 2023. Column (1) presents univariate regression results, controlling for *year* and *industry* fixed effects. Column (2) reports multivariate results that control for firm-level characteristics. t-statistics are reported in parentheses. ^***^, ^**^, and ^*^ indicate statistical significance at the 1%, 5%, and 10% levels, respectively.

Turning to the control variables, the positive and significant coefficient on *Size* suggests that larger firms may face greater opportunities or pressures to engage in earnings management. In contrast, *Roa* is negatively associated with *REM*, implying that firms with stronger profitability are less inclined toward earnings manipulation. *Growth* exhibits a significantly positive relationship with *REM*, indicating that firms experiencing rapid revenue growth may resort to earnings management to meet market expectations.

Regarding governance-related variables, *Dual* shows a negative coefficient, suggesting that CEO duality may enhance oversight effectiveness and curb opportunistic behavior. The presence of a Big Four auditor (*Big4*), a higher proportion of independent directors (*Indep*), and a larger board size (*Board*) are all significantly associated with lower levels of REM. These findings highlight the pivotal role of corporate governance in curbing REM activities. Finally, the significantly positive coefficient on *AEM* suggests that firms tend to employ both accrual-based and REM techniques simultaneously.

### 4.3 Endogeneity tests

#### 4.3.1 Instrumental variable approach.

To address potential endogeneity concerns arising from omitted variable bias and reverse causality, we employ an instrumental variable (IV) approach. Following Huang et al. (2019) and Nunn and Qian (2014) [39,40], we construct the instrumental variable by interacting the number of fixed-line telephones per 100 people in the city where the firm is located in the current year with the number of internet users in the previous year.

Regarding relevance, fixed-line telephones represent the foundational telecommunications infrastructure, while the number of internet users reflects the regional maturity of digital adoption. The interaction of these two factors captures the comprehensive external environment for digital resource aggregation. A region with robust infrastructure and high internet penetration significantly lowers the costs of data acquisition and processing, thereby facilitating corporate data asset accumulation.

Regarding exogeneity, this instrument meets the exclusion restriction for two reasons. First, as a regional macroeconomic indicator determined by historical aggregate demand and urban planning, the number of telephones is exogenous to any individual firm’s specific REM decisions, thereby ruling out reverse causality. Second, the use of a lagged term for internet users effectively severs the link with current-period shocks to firm performance or financial reporting behavior, mitigating concerns regarding simultaneity bias.

The results are presented in columns (1) and (2) of Table 4. In the first stage of the regression, *DA* is the dependent variable. Results indicate that the coefficient for the instrument variable is positive and significant. In the second step regression, *REM* is the dependent variable. Results show that the instrumented *DA* coefficient is still negative and significant (−0.171, t = −5.795). The results indicate that after correction for possible endogeneity bias, the data source continues to have a significant *REM* inhibitory effect, in line with our baseline findings.

**Table 4:** Endogeneity tests.

Variables	(1)	(2)	(3)	(4)	(5)	(6)	(7)
	First stage	Second stage	PSM	Cluster by firm	Cluster by region	Cluster by region-year	Cluster by firm-year
	*DA*	*REM*	*REM*	*REM*	*REM*	*REM*	*REM*
*DA*		-0.171^***^	-0.019***	-0.017***	-0.017**	-0.017***	-0.017***
		(-5.795)	(-6.284)	(-4.232)	(-5.065)	(-6.665)	(-7.595)
*DA_IV*	0.000^***^						
	(11.015)						
*Size*	0.005	0.029^***^	0.030^***^	0.030^***^	0.030^***^	0.030^***^	0.030^***^
	(1.076)	(15.683)	(13.831)	(9.639)	(26.423)	(19.723)	(19.275)
*Lev*	-0.236^***^	-0.024^*^	0.017	0.015	0.015	0.015	0.015
	(-7.735)	(-1.750)	(1.339)	(0.945)	(1.047)	(0.955)	(1.601)
*Roa*	-1.018^***^	-2.457^***^	-2.288^***^	-2.295^***^	-2.294^***^	-2.294^***^	-2.295^***^
	(-10.158)	(-47.562)	(-47.636)	(-40.055)	(-69.668)	(-24.186)	(-66.131)
*Growth*	0.086^***^	0.091^***^	0.086^***^	0.077^***^	0.077^***^	0.077^***^	0.077^***^
	(7.004)	(12.678)	(9.944)	(11.548)	(12.006)	(5.153)	(12.680)
*Dual*	0.039^***^	-0.006	-0.016^***^	-0.013^**^	-0.013^*^	-0.013^***^	-0.013^***^
	(3.976)	(-1.475)	(-4.039)	(-2.528)	(-3.400)	(-6.108)	(-4.513)
*Soe*	-0.104^***^	-0.011^**^	-0.001	0.002	0.002	0.002	0.002
	(-8.841)	(-2.095)	(-0.197)	(0.249)	(0.210)	(0.585)	(0.533)
*Top1*	-0.002^***^	-0.000^**^	-0.000	-0.000	-0.000^**^	-0.000^**^	-0.000^*^
	(-6.396)	(-2.293)	(-0.606)	(-0.923)	(-7.866)	(-2.039)	(-1.863)
*Big4*	-0.065^***^	-0.057^***^	-0.049^***^	-0.047^***^	-0.047^**^	-0.047^***^	-0.047^***^
	(-4.205)	(-9.355)	(-6.819)	(-4.184)	(-5.856)	(-10.561)	(-9.005)
*Indep*	0.004^***^	0.000	-0.001^***^	-0.001	-0.001	-0.001^***^	-0.001^***^
	(4.064)	(0.114)	(-2.956)	(-1.608)	(-1.772)	(-3.032)	(-2.923)
*Board*	0.002^***^	0.000^***^	-0.027^**^	-0.027^*^	-0.026^**^	-0.026^***^	-0.027^***^
	(7.539)	(2.661)	(-2.458)	(-1.753)	(-5.536)	(-4.187)	(-3.293)
*Mshare*	-0.030	-0.028^***^	0.000	0.000	0.000	0.000	0.000
	(-1.132)	(-2.933)	(0.241)	(0.224)	(0.467)	(0.383)	(0.456)
*Mpay*	0.050^***^	-0.031^***^	-0.044^***^	-0.043^***^	-0.043^**^	-0.043^***^	-0.043^***^
	(6.475)	(-9.144)	(-12.935)	(-8.784)	(-7.729)	(-15.589)	(-17.184)
*AEM*	-0.034	1.071^***^	1.046^***^	1.054^***^	1.053^***^	1.053^***^	1.054^***^
	(-0.652)	(47.465)	(38.004)	(44.974)	(20.602)	(10.232)	(53.234)
Constant	2.545^***^	0.432^***^	0.111*	0.068	0.067	0.067	0.068
	(18.119)	(4.956)	(1.874)	(0.830)	(0.694)	(1.466)	(1.578)
N	17940	17940	10907	20770	20770	20770	20770
Adj. R2	0.425	0.240	0.388	0.387	0.387	0.387	0.387
K-P rk LM	119.42***						
K-P rk Wald F	121.33***						
Year FE	YES	YES	YES	YES	YES	YES	YES
Industry FE	YES	YES	YES	YES	YES	YES	YES

Note: This table presents the results of endogeneity tests. Columns (1) and (2) report the two-stage least squares (2SLS) estimation results using the instrumental variable (IV) approach, where the IV is constructed as the interaction term between the number of fixed-line telephones in the firm’s city and the lagged number of national internet users. The reduced sample size in Columns (1) and (2) compared to the baseline sample is due to missing historical city-level telecommunications data required to construct the instrumental variable. Column (3) reports the results based on the propensity score matching (PSM) approach, using 1:1 nearest-neighbor matching without replacement. Columns (4) to (7) display the regression results under alternative clustering methods, with standard errors clustered by firm, region, firm-year and region-year, respectively. t-statistics are reported in parentheses. ^***^, ^**^, and ^*^ denote statistical significance at the 1%, 5%, and 10% levels, respectively.

#### 4.3.2 Propensity score matching.

To address potential endogeneity arising from self-selection, we implement a propensity score matching (PSM) approach. Firms with data asset levels above the sample median are classified as the treatment group, while those below the median constitute the control group. Propensity scores are estimated using a logit model based on the following covariates: *Size*, *Lev*, *Roa*, *Growth*, *Board*, *Age* (measured as the number of years since listing), and *FC* (financial constraint, measured using the FC index). We apply 1:1 nearest-neighbor matching without replacement.

The post-matching covariate balance test, presented in Table 5, indicates that most covariates achieve satisfactory balance. While there remains a marginal difference in *Size* between the treatment and control groups, the overall matching significantly reduces the differences across the covariates. Re-estimating the regression model using the matched sample, as reported in Column (3) of Table 4, we find that the coefficient on *DA* remains significantly negative (−0.019, t = −6.284), further reinforcing the robustness of our main findings.

**Table 5:** Covariate balance test.

Variable	Unmatched/Matched	Treated	Control	%Bias	t	p > |t|
*Size*	U	22.423	22.686	−20.7	−14.81	0.000
	M	22.424	22.455	−2.5	−1.79	0.074
*Lev*	U	0.40729	0.44834	−21.3	−15.22	0.000
	M	0.40747	0.40906	−0.8	−0.60	0.552
*Roa*	U	0.05079	0.05263	−3.3	−2.33	0.020
	M	0.05098	0.05198	−1.8	−1.26	0.206
*Growth*	U	0.20929	0.16987	11.0	7.89	0.000
	M	0.20795	0.20913	−0.3	−0.22	0.826
*Board*	U	2.1148	2.1528	−19.5	−13.99	0.000
	M	2.115	2.1151	−0.1	−0.04	0.972
*Age*	U	2.0918	2.3374	−36.1	−25.83	0.000
	M	2.093	2.0869	0.9	0.63	0.531
*FC*	U	0.46572	0.4032	22.8	16.31	0.000
	M	0.46557	0.45984	2.1	1.48	0.139

Note: This table reports the covariate balance test results before and after propensity score matching (PSM). The sample is divided into treated and control groups based on whether firms’ data asset levels are above or below the sample median. The test compares the means of each covariate between the treated and control groups in both the unmatched (U) and matched (M) samples. %Bias represents the standardized difference in means, while t- and p-values are based on two-sample t-tests for equality of means.

#### 4.3.3 Alternative clustering methods.

To address potential cross-sectional correlations at the firm and regional levels, we cluster standard errors by firm, region, region-year and firm-year, respectively. Columns (4) to (7) of Table 4 report the regression results under these alternative clustering methods. Across all specifications, the coefficient on *DA* consistently remains −0.017 and is statistically significant at either the 1% or 5% level, further strengthening the robustness of our main findings.

### 4.4 Other robustness tests

#### 4.4.1 Alternative samples.

To ensure the robustness of our findings, we conducted two tests by altering the sample composition. First, we excluded observations prior to 2013, focusing on a period characterized by rapid internet development and widespread adoption of smart devices in China. Since 2013, technological advancements have triggered an exponential growth in data generation, prompting firms to invest more heavily in data assets to enhance management and operational efficiency. This period also coincides with the launch of China’s National Broadband Strategy, which aimed to expand broadband infrastructure and accelerate 4G network coverage nationwide. By restricting the sample to the post-2013 period, we better capture an environment where data assets became increasingly critical to corporate operations.

Second, we exclude industries with inherent data advantages to mitigate potential bias. Firms in the information or communication sectors naturally generate and process large volumes of data, granting them a distinct advantage in accumulating and leveraging data assets. To address this concern, we exclude all firms classified under the Information Transmission, Software, and Information Technology Services sectors.

Columns (1) and (2) of Table 6 present the results based on these alternative samples. In both cases, the coefficient on *DA* remains significantly negative at the 1% level, further strengthening the robustness of our main findings.

**Table 6:** Robustness tests.

Variable	(1)	(2)	(3)	(4)
	*REM*	*REM*	*REM*	*REM*
*DA*	−0.017^***^	−0.017^***^		
	(−7.226)	(−7.170)		
*DA_1*			−2.816^***^	
			(−3.487)	
*DA_2*				−0.016^***^
				(−7.279)
*Size*	0.031^***^	0.031^***^	0.030^***^	0.030^***^
	(18.586)	(18.927)	(19.236)	(19.236)
*Lev*	0.025^**^	0.013	0.018^**^	0.015
	(2.540)	(1.297)	(1.979)	(1.628)
*Roa*	−2.201^***^	−2.308^***^	−2.280^***^	−2.294^***^
	(−60.676)	(−62.363)	(−65.588)	(−66.117)
*Growth*	0.085^***^	0.081^***^	0.075^***^	0.077^***^
	(13.513)	(12.936)	(12.396)	(12.670)
*Dual*	−0.013^***^	−0.013^***^	−0.014^***^	−0.014^***^
	(−4.230)	(−4.315)	(−4.733)	(−4.535)
*Soe*	0.006^*^	0.001	0.003	0.002
	(1.669)	(0.320)	(0.991)	(0.554)
*Top1*	−0.000^**^	−0.000^**^	−0.000	−0.000^*^
	(−2.147)	(−2.514)	(−1.610)	(−1.861)
*Big4*	−0.048^***^	−0.043^***^	−0.046^***^	−0.047^***^
	(−8.894)	(−8.169)	(−8.800)	(−8.991)
*Indep*	−0.001^***^	−0.001^***^	−0.001^***^	−0.001^***^
	(−3.246)	(−3.049)	(−3.200)	(−2.936)
*Board*	−0.032^***^	−0.035^***^	−0.027^***^	−0.027^***^
	(−3.708)	(−4.230)	(−3.299)	(−3.295)
*Mshare*	−0.000	0.000	0.000	0.000
	(−0.733)	(0.149)	(0.108)	(0.446)
*Mpay*	−0.045^***^	−0.041^***^	−0.044^***^	−0.043^***^
	(−16.874)	(−16.189)	(−17.630)	(−17.183)
*AEM*	1.005^***^	1.030^***^	1.054^***^	1.054^***^
	(48.129)	(50.627)	(53.188)	(53.275)
Constant	0.127^***^	0.062	0.028	0.016
	(2.747)	(1.424)	(0.656)	(0.372)
N	17879	19402	20770	20770
Adj. R2	0.383	0.388	0.386	0.387
Year FE	YES	YES	YES	YES
Industry FE	YES	YES	YES	YES

Note: This table presents the results of robustness tests using alternative sample compositions and alternative measures of data assets. Column (1) excludes observations prior to 2013 to focus on the period following the launch of China’s National Broadband Strategy, during which the development and application of data assets accelerated. Column (2) excludes firms operating in the Information Transmission, Software, and Information Technology Services industries. Columns (3) and (4) assess the robustness of our findings using alternative measures of data assets. In Column (3), we use “data assets” and “data resources” as seed words and apply a Word2Vec-based approach to identify semantically similar terms. *DA_1* is defined as the frequency of these dictionary words in each firm’s annual report divided by the total word count. In Column (4), *DA_2* is a relative measure, calculated as a firm’s data assets minus the corresponding industry-year average. t-statistics are reported in parentheses. ^***^, ^**^, and ^*^ denote statistical significance at the 1%, 5%, and 10% levels, respectively.

#### 4.4.2 Changing the measurement of data assets.

We adopt an alternative approach following the methodology of Sun and Du (2024) [3]. Specifically, we select “data assets” and “data resources” as seed words and apply deep learning techniques using the Word2Vec neural network model to identify semantically similar terms. The top ten words with the highest similarity scores are retained to construct an expanded data asset dictionary. We then define an alternative measure, *DA_1*, as the frequency of these dictionary words in each firm’s annual report divided by the total word count, as specified by the following equation:


$$\begin{array}{c} {DA}_{it}=\sum Dictionary\;Words_{itn}/TotalWords_{it}*\text{1}\text{0}\text{0} \end{array}$$ (6)

where *Dictionary Word*_*itn*_ is the frequency of the n-th dictionary word in firm *i*’s annual report in year *t*, and *Total Words*_*it*_ is the total word count of the annual report.

In addition, we construct a relative measure, *DA_2*, defined as the difference between a firm’s data assets and the corresponding industry-year average. This variable captures the firm’s data asset intensity relative to its industry peers. The regression results, presented in Columns (3) and (4) of Table 6, show that both *DA_1* and *DA_2* exhibit significantly negative coefficients at the 1% level, further strengthening the robustness of our main findings.

## 5 Mechanism analysis

### 5.1 Managerial short-termism

Building on the preceding hypothesis, we propose that data assets help mitigate REM by reducing managerial short-termism. Short-termism refers to a managerial bias toward achieving immediate financial outcomes at the expense of long-term value creation [41]. Driven by market pressures and incentive structures, such myopic behavior can be moderated by factors that enhance governance quality or extend managerial time horizons, thereby reducing reliance on earnings manipulation [35].

Data assets play a critical role in curbing managerial short-termism. First, data assets enhance internal controls and improve information transparency. By leveraging big data resources, comprehensive training datasets, and advanced recognition algorithms, firms can generate more accurate earnings forecasts and provide real-time information to stakeholders [42]. This increased transparency raises the cost of opportunistic manipulation by amplifying the risk of detection, thereby discouraging myopic behavior. Moreover, enhanced informational clarity alleviates the uncertainty encountered by managers, which constitutes a key driver of short-term decision-making under performance pressure [43].

Second, data assets help broaden managerial time horizons. By integrating key business dimensions—such as production efficiency, customer engagement, market pricing, and sales performance—into centralized, data-driven systems, firms gain a comprehensive view of their operations. Machine learning models and predictive analytics highlight long-term growth opportunities and guide more efficient resource allocation, aligning firm performance with sustainable value creation [44]. In such a data-intensive ecosystem, the marginal benefit of engaging in REM diminishes, as decisions become increasingly evidence-based rather than tactic-driven. Consequently, managers are incentivized to prioritize durable performance outcomes over short-term financial optics.

To empirically examine whether data assets mitigate REM by reducing managerial short-termism, we construct a Myopia Index through textual analysis of the Management’s Discussion and Analysis (MD&A) sections in annual reports. Drawing on the short-term orientation lexicon developed by Brochet et al. (2015) [45], we adapt and expand this word list to better align with the Chinese linguistic and business context. First, we manually select a set of seed words categorized into direct expressions (e.g., “within days,” “several months,” “immediately,” “at once”) and indirect expressions (e.g., “opportunity,” “occasion,” “pressure,” “challenge”) that subtly imply a short-term mindset. Next, we employ the Continuous Bag-of-Words (CBOW) model within the Word2Vec framework to train word embeddings on a corpus of annual financial reports. By calculating vector similarities, we identify additional words closely related to our seed terms, ultimately forming an expanded vocabulary representing short-term orientation. Finally, we compute the Myopia Index (*Short-termism*) as the frequency of short-term-oriented words divided by the total word count of the MD&A section, multiplied by 100. A higher index value indicates a greater degree of managerial short-termism.

Columns (1) and (2) of Table 7 present the baseline mechanism analysis using the text-based Myopia Index. In Column (1), where managerial *Short-termism* is the dependent variable, the coefficient on *DA* is significantly negative (−0.009, t = −20.153), suggesting that firms with higher levels of data assets tend to exhibit a lower degree of short-term orientation. In Column (2), with *REM* as the dependent variable, *DA* remains significantly negative (−0.016, t = −7.000), while *Short-termism* enters with a significantly positive coefficient (0.126, t = 3.354). This positive association indicates that greater managerial myopia is indeed a driver of REM.

**Table 7:** Mechanism analysis.

Variable	(1)	(2)	(3)	(4)	(5)	(6)
	*Short-termism*	*REM*	*Shortinv*	*REM*	*Analyst coverage*	*REM*
*DA*	−0.009^***^	−0.016^***^	−0.004^***^	−0.016^***^	0.093^***^	−0.014^***^
	(−20.153)	(−7.000)	(−4.038)	(−7.338)	(9.315)	(−6.414)
*Short-termism*		0.126^***^				
		(3.354)				
*Shortinv*				0.046^***^		
				(2.621)		
*Analyst coverage*						−0.028^***^
						(−16.934)
*Size*	0.001^***^	0.030^***^	0.002^***^	0.030^***^	0.345^***^	0.040^***^
	(3.140)	(19.227)	(2.927)	(19.230)	(53.246)	(23.712)
*Lev*	0.007^***^	0.014	−0.065^***^	0.015^*^	−0.218^***^	0.009
	(4.060)	(1.511)	(−19.985)	(1.657)	(−5.471)	(0.950)
*Roa*	−0.031^***^	−2.291^***^	0.097^***^	−2.274^***^	5.794^***^	−2.131^***^
	(−6.300)	(−66.000)	(8.242)	(−66.001)	(43.538)	(−60.336)
*Growth*	−0.004^***^	0.077^***^	0.001	0.083^***^	−0.007	0.076^***^
	(−6.991)	(12.732)	(0.888)	(14.081)	(−0.403)	(12.530)
*Dual*	−0.002^***^	−0.013^***^	0.004^***^	−0.013^***^	0.093^***^	−0.011^***^
	(−4.968)	(−4.412)	(3.498)	(−4.529)	(7.372)	(−3.664)
*Soe*	0.006^***^	0.001	−0.001	−0.000	−0.176^***^	−0.003
	(9.126)	(0.313)	(−1.223)	(−0.049)	(−11.990)	(−0.951)
*Top1*	0.000	−0.000^*^	0.000^**^	−0.000	−0.003^***^	−0.000^***^
	(1.270)	(−1.892)	(2.264)	(−1.514)	(−8.023)	(−2.849)
*Big4*	0.002^**^	−0.047^***^	−0.000	−0.046^***^	0.020	−0.046^***^
	(2.151)	(−9.064)	(−0.139)	(−9.053)	(0.944)	(−8.959)
*Indep*	0.000	−0.001^***^	−0.000	−0.001^***^	0.003^***^	−0.001^***^
	(0.901)	(−2.944)	(−1.069)	(−3.037)	(2.908)	(−2.587)
*Board*	0.001	−0.027^***^	0.000^***^	0.000	0.067^*^	−0.025^***^
	(0.927)	(−3.319)	(4.254)	(0.063)	(1.904)	(−3.085)
*Mshare*	−0.000^***^	0.000	−0.014^***^	−0.023^***^	0.004^***^	0.000^*^
	(−6.128)	(0.581)	(−4.849)	(−2.919)	(10.269)	(1.688)
*Mpay*	−0.000	−0.043^***^	0.000	−0.042^***^	0.169^***^	−0.038^***^
	(−1.001)	(−17.175)	(0.133)	(−17.159)	(16.491)	(−15.387)
*AEM*	−0.008^***^	1.055^***^	−0.010^*^	1.044^***^	−0.065	1.052^***^
	(−2.606)	(53.253)	(−1.840)	(53.456)	(−0.947)	(53.405)
Constant	0.053^***^	0.061	0.055^***^	0.053	−8.123^***^	−0.162^***^
	(6.628)	(1.419)	(3.498)	(1.250)	(−45.021)	(−3.596)
N	20770	20770	20655	20655	20770	20770
Adj. R2	0.150	0.388	0.117	0.389	0.344	0.396
Year FE	YES	YES	YES	YES	YES	YES
Industry FE	YES	YES	YES	YES	YES	YES

Note: This table presents the results of the mechanism analysis examining the mediating roles of managerial short-termism and analyst coverage in the relationship between data assets and REM. Columns (1) and (2) test the short-termism channel, while Columns (3) and (4) test the analyst coverage channel. Short-termism is measured using a text-based approach that captures the frequency of short-term-oriented keywords in the Management’s Discussion and Analysis (MD&A) sections of annual reports. Analyst coverage is measured as the natural logarithm of the number of financial analysts following a firm in a given year. t-statistics are reported in parentheses. ^***^, ^**^, and ^*^ denote statistical significance at the 1%, 5%, and 10% levels, respectively.

To validate these findings and address potential concerns regarding the subjectivity of textual analysis, we introduce an objective, asset-based proxy for short-termism. Drawing on the work of Demir (2009) [46], we construct *Shortinv*, defined as the proportion of short-term investments (trading financial assets, available-for-sale financial assets, and held-to-maturity investments) to total assets. A higher ratio implies a managerial preference for liquidity and short-term financial performance over long-term value creation. Columns (3) and (4) report the results of this validation test. In Column (3), the coefficient on *DA* is significantly negative (−0.004, t = −4.038), indicating that data assets are associated with a reduction in the allocation of short-term assets. Furthermore, in Column (4), *Shortinv* is positively associated with *REM* (0.046, t = 2.621), while the negative effect of *DA* on *REM* remains robust (−0.016, t = −7.338).

Taken together, results from both the text-based and asset-based measures provide consistent empirical support for the proposed mechanism: data assets mitigate REM by curbing managerial short-termism.

### 5.2 Analyst coverage

A firm’s proactive disclosure of data assets conveys a strong strategic signal to external stakeholders, highlighting its capacity to manage data as a core organizational resource [29]. Prior research suggests that detailed and comprehensive corporate disclosures attract greater analyst coverage by reducing information asymmetry and enhancing firm transparency [47]. In the context of the digital economy, where data assets are increasingly recognized as critical drivers of long-term value, such disclosures provide analysts with valuable inputs for assessing firm-specific risks, opportunities, and strategic capabilities.

Financial analysts serve as influential external monitors within capital markets, and their continuous scrutiny imposes market discipline on corporate managers [48]. Firms subject to more intensive analyst coverage face greater accountability for financial reporting quality, as analysts not only disseminate firm-specific information but also act as gatekeepers for investors and regulators. Empirical evidence confirms that firms under heightened analyst scrutiny are less likely to engage in opportunistic earnings manipulation due to improved transparency and external monitoring pressure [38]. Consequently, we posit that the disclosure of data assets attracts increased analyst attention, which in turn constrains managers’ propensity to engage in REM.

To empirically test the proposed mechanism, we construct the variable *Analyst coverage*, measured as the natural logarithm of the number of financial analysts following a firm. Columns (3) and (4) of Table 7 present the results of the mechanism analysis investigating whether analyst coverage mediates the relationship between data assets and REM. In Column (3), the regression results show that *DA* has a significantly positive effect on *Analyst coverage* (0.093, t = 9.315), indicating that firms with greater engagement in data assets attract more attention from financial analysts. In Column (4), where *REM* is the dependent variable, *DA* continues to exhibit a significantly negative coefficient (−0.014, t = −6.414), while *Analyst coverage* also shows a significant and negative association with *REM* (−0.028, t = −16.934). These findings suggest that analyst coverage acts as a mediating channel through which data assets reduce REM, providing further empirical support for the proposed mechanism.

## 6 Further analysis

### 6.1 Moderating effect of data assets on real earnings management under earnings pressure

We further investigate the moderating effect of data assets on REM in the presence of earnings pressure. Specifically, we focus on situations where firms fail to meet analyst consensus earnings forecasts, a widely used proxy for capital market performance pressure. Firms that fall short of market expectations are more likely to experience negative investor reactions and thus face stronger incentives to engage in earnings manipulation [49]. However, data assets can help mitigate such opportunistic behavior by enhancing internal controls and improving transparency [4, 9]. Accordingly, the governance effect of data assets is expected to be more pronounced among firms facing greater earnings pressure.

To empirically test this expectation, we introduce an interaction term between *DA* and an indicator variable (*Miss_forecast*) that equals one if the firm fails to meet the analyst consensus earnings forecast. The regression results, presented in Column (1) of Table 8, show that the coefficient on *Miss_forecast* is significantly positive (0.037, t = 2.679), indicating that firms missing market expectations are more likely to engage in REM. More importantly, the significantly negative coefficient on the interaction term Miss_forecast*DA (−0.012, *t* = −2.802) indicates that data assets weaken the positive impact of missing analyst forecasts on REM. In other words, the presence of stronger data assets mitigates the tendency of firms under earnings pressure to engage in REM.

**Table 8:** Further analysis.

Variable	(1)	(2)	(3)	(4)	(5)	(6)
	*REM*	*Abprod*	*Abcfo*	*Abdisx*	*Tobin’s Q*	*Tobin’s Q*
*DA*	−0.008^**^	−0.011^***^	−0.003^***^	0.009^***^	0.055^***^	0.042^***^
	(−2.175)	(−6.972)	(−3.387)	(8.984)	(3.467)	(2.678)
*Miss_forecast*	0.037^***^					
	(2.679)					
Miss_forecast*DA	−0.012^***^					
	(−2.802)					
*REM*						−0.758^***^
						(−11.848)
*Size*	0.030^***^	0.019^***^	0.001^**^	−0.015^***^	−0.433^***^	−0.410^***^
	(19.212)	(8.880)	(2.208)	(−20.884)	(−32.752)	(−30.598)
*Lev*	0.014	0.013	−0.029^***^	0.036^***^	−0.329^***^	−0.318^***^
	(1.544)	(1.332)	(−7.587)	(8.251)	(−4.455)	(−4.349)
*Roa*	−2.300^***^	−1.214^***^	0.966^***^	0.310^***^	7.047^***^	5.302^***^
	(−65.125)	(−34.239)	(75.226)	(19.467)	(23.294)	(17.320)
*Growth*	0.076^***^	0.030^***^	−0.024^***^	−0.016^***^	0.081^***^	0.139^***^
	(12.614)	(2.876)	(−6.284)	(−5.218)	(3.115)	(5.117)
*Dual*	−0.013^***^	−0.006^**^	0.001	0.006^***^	0.085^***^	0.074^***^
	(−4.427)	(−2.502)	(0.808)	(4.568)	(3.744)	(3.306)
*Soe*	0.002	−0.001	−0.000	−0.001	0.014	0.016
	(0.500)	(−0.467)	(−0.402)	(−0.553)	(0.607)	(0.663)
*Top1*	−0.000^*^	−0.000	0.000	0.000^**^	−0.001^*^	−0.001^**^
	(−1.887)	(−0.531)	(0.126)	(2.253)	(−1.936)	(−2.139)
*Big4*	−0.047^***^	−0.028^***^	0.003^**^	0.020^***^	0.247^***^	0.211^***^
	(−8.965)	(−7.063)	(2.071)	(8.196)	(6.067)	(5.193)
*Indep*	−0.001^***^	−0.000	0.000	0.000^**^	0.008^***^	0.007^***^
	(−2.920)	(−1.483)	(1.386)	(2.263)	(3.789)	(3.486)
*Board*	−0.027^***^	−0.007	0.004	0.008^**^	−0.098	−0.119^*^
	(−3.330)	(−1.143)	(1.564)	(2.228)	(−1.510)	(−1.843)
*Mshare*	0.000	−0.000	−0.000^**^	0.000	−0.010^***^	−0.010^***^
	(0.483)	(−0.447)	(−2.324)	(0.585)	(−15.183)	(−15.232)
*Mpay*	−0.043^***^	−0.023^***^	0.000	0.022^***^	0.143^***^	0.111^***^
	(−17.164)	(−7.875)	(0.087)	(18.675)	(8.696)	(6.735)
*AEM*	1.054^***^	0.342^***^	−0.710^***^	−0.115^***^	−0.651^***^	0.156
	(53.142)	(14.265)	(−61.910)	(−12.294)	(−5.367)	(1.188)
Constant	0.042	−0.026	−0.014	−0.058^***^	9.909^***^	9.959^***^
	(0.956)	(−0.807)	(−0.919)	(−3.234)	(27.280)	(27.627)
N	20770	20770	20770	20770	20770	20770
Adj. R2	0.387	0.200	0.538	0.095	0.303	0.310
Year FE	YES	YES	YES	YES	YES	YES
Industry FE	YES	YES	YES	YES	YES	YES

Note: This table presents further analyses on the role of data assets in mitigating REM. Column (1) examines the moderating effect of *DA* under earnings pressure, measured by an indicator variable *Miss_forecast*, which equals one if a firm fails to meet the analyst consensus earnings forecast. The interaction term Miss_forecast*DA is included to test whether data assets mitigate the impact of missing analyst forecasts on REM. Columns (2) to (4) explore how data assets relate to different components of REM, including abnormal production costs (*Abprod*), abnormal cash flow from operations (*Abcfo*), and abnormal discretionary expenses (*Abdisx*). Columns (5) and (6) assess whether data assets contribute to enhan9999ced long-term performance through the channel of reduced REM, using *Tobin’s Q* as the outcome variable. t-statistics are reported in parentheses. ^***^, ^**^, and ^*^ denote statistical significance at the 1%, 5%, and 10% levels, respectively.

### 6.2 Impact of data assets on components of real earnings management

This subsection examines how data assets affect individual components of REM, namely abnormal production costs (*Abprod*), abnormal operating cash flows (*Abcfo*), and abnormal discretionary expenses (*Abdisx*). By disaggregating REM into these three components, we aim to provide a more nuanced understanding of the channels through which data assets influence managerial financial reporting behavior. Theoretically, we expect a negative relationship between *DA* and *Abprod*, indicating reduced manipulation of production costs. Conversely, we anticipate positive relationships between *DA* and both *Abcfo* and *Abdisx*, reflecting reduced manipulation in cash flows and discretionary expenses, respectively.

Columns (2), (3), and (4) of Table 8 present the regression results. In Column (2), the coefficient of *DA* on *Abprod* is −0.011 (t = −6.972), consistent with the expectation of reduced production cost manipulation. In Column (3), the coefficient of *DA* on *Abcfo* is −0.003 (t = −3.387), which contradicts the expected positive relationship. In Column (4), the coefficient of *DA* on *Abdisx* is 0.009 (t = 8.984), significant at the 1% level, aligning with the theoretical prediction. These findings suggest that data assets primarily mitigate REM by reducing the propensity for cost-side operational manipulation, particularly in production activities and discretionary expenditures. However, the observed negative association between *DA* and *Abcfo* introduces an intriguing nuance. This result may reflect structural changes in operational cash flow patterns, potentially driven by more data-informed sales strategies or the adoption of more conservative revenue recognition practices.

### 6.3 The effect of data assets on long-term performance through reduced real earnings management

REM is often employed to inflate reported earnings by manipulating operational activities. However, such practices can undermine investment efficiency and distort resource allocation, ultimately impairing long-term firm value [21]. This subsection examines whether data assets enhance long-term firm performance by mitigating REM. We use *Tobin’s Q* as a forward-looking measure of firm value, capturing investors’ expectations regarding the firm’s growth potential.

Columns (5) and (6) of Table 8 report the regression results. In Column (5), the coefficient on *DA* is significantly positive (0.055, t = 3.467), suggesting that firms with greater engagement in data assets tend to exhibit higher long-term performance. In Column (6), after controlling for *REM*, the coefficient on *DA* remains significantly positive (0.042, t = 2.678), while the coefficient on *REM* is significantly negative (−0.758, t = −11.848). These findings indicate that data assets not only directly contribute to long-term value creation but also indirectly enhance long-term performance by mitigating REM.

## 7 Conclusions

This study examines whether data assets can mitigate REM. Using a panel of 20,770 firm-year observations from A-share listed companies in China between 2010 and 2023, we document a significantly negative association between data assets and REM, which remains robust across multiple endogeneity and robustness tests.

Our mechanism analysis sheds light on the underlying processes driving this relationship: data assets curb REM by weakening managerial short-termism and by attracting greater attention from financial analysts, thereby enhancing both internal discipline and external oversight. Further analyses reveal that the mitigating effect of data assets on REM is particularly pronounced among firms facing earnings pressure, especially those failing to meet analyst forecasts. Moreover, our results show that data assets primarily mitigate REM by reducing manipulation in production costs and discretionary expenses, rather than by influencing operating cash flows. Finally, we find that the reduction of REM through the use of data assets contributes to enhanced long-term firm performance.

Our findings offer several important practical implications. For firms, they underscore the strategic value of investing in data infrastructure, not only for operational efficiency but also as a component of a robust governance framework. For policymakers, our results suggest that promoting standardized and transparent data assets reporting can strengthen external governance. This aligns with recent policy initiatives, such as China’s 2023 *Interim Provisions on Accounting Treatment of Enterprise Data Resources*. By formalizing data as a balance-sheet asset, this policy is likely to amplify the transparency and monitoring effects we document. In jurisdictions without such mandatory disclosure requirements, our findings imply that voluntary disclosure of data assets can serve as a credible signal of governance quality, allowing high-quality firms to distinguish themselves. Furthermore, the governance mechanisms identified in this study are likely to be strengthened by the adoption of AI-driven audit tools. As noted in prior literature, technologies that enable continuous, full-population transaction analysis can leverage corporate data assets to automate the detection of operational anomalies in real time. This technological synergy significantly raises the detection risk for managers attempting to conceal REM. Finally, from a strategic perspective, the deployment of data assets should be viewed not merely as a compliance exercise but as a value-enhancing initiative that fosters financial discipline and sustainable competitive advantage.

This study is subject to several limitations. First, its focus on a single country means the findings may not be fully generalizable to economies with different institutional environments, legal frameworks, and levels of digital development. Second, our measure of data assets relies on a textual-frequency proxy. While grounded in established methods, this approach captures the disclosure of data assets and may not perfectly reflect the quality or efficiency of a firm’s actual data utilization capabilities. Future research could address these limitations through cross-country comparative studies to examine how varying institutional factors moderate the governance role of data assets.

## Supporting information

S1 Table The Dictionary of Data Asset Keywords. (XLSX)
